# Improved Expansion and *In Vivo* Function of Patient T Cells by a Serum-free Medium

**DOI:** 10.1016/j.omtm.2017.11.001

**Published:** 2017-12-14

**Authors:** Andrew R. Medvec, Christopher Ecker, Hong Kong, Emily A. Winters, Joshua Glover, Angel Varela-Rohena, James L. Riley

**Affiliations:** 1 Department of Microbiology and Center for Cellular Immunotherapies, University of Pennsylvania, Philadelphia, PA 19104, USA; 2 Gibco BioProduction Cell Culture and Cell Therapy, Thermo Fisher Scientific, 3175 Staley Road, Grand Island, NY 14072, USA

**Keywords:** adoptive T cell therapy, leukemia, effector memory T cell, patient T cell

## Abstract

Improvements to T cell culture systems that promote long-term engraftment and function of adoptively transferred T cells will likely result in superior clinical benefit to more individuals. To this end, we recently developed a chemically defined cell culture medium that robustly expands all T cell subsets in the absence of human serum. Using a humanized mouse model, we observed that T cells expanded in the absence of human serum provided durable control of tumors, whereas T cells expanded in medium supplemented with human serum only mediated transient control of tumor growth. Importantly, our new medium effectively expanded more differentiated T cells from multiple myeloma patients in the absence of serum. These patient-derived T cells were also able to provide durable control of B cell tumors *in vivo*, and this long-term control of cancer was lost when T cells were expanded in the presence of serum. Thus, engineered T cells expanded in an optimized medium in the absence of serum may have improved therapeutic potential.

## Introduction

Adoptive transfer of T cells re-directed to tumor-specific antigens by genetic engineering has shown great promise to treat, and, in some cases, cure immune-cell-based cancers (reviewed in Sadelain,^1^ Rosenberg and Restifo,^2^ and Barrett^43^). In a recent study, infusion of up to 2 × 10^7^ T cells/kg engineered to express a chimeric antigen receptor (CAR) linking a single chain antibody specific for human CD19 to a signaling complex composed of the 4-1BB and CD3 zeta cytoplasmic domains was able to provide durable complete remissions to 90% of patients suffering from relapsed or refractory acute B lymphoblastic leukemia (B-ALL).^3^ However, 24% of the patients were not able to receive this therapy because their T cells failed to expand adequately *ex vivo* and the target dose was not achieved.^4^ This highlights the difficulty of expanding T cells from cancer patients and the need to develop more efficient ways to manufacture T cells for adoptive T cell therapy. Additionally, long-term persistence of functional engineered T cells is key to success of these therapies. Porter et al.^5^ observed a strong correlation between T cell persistence and improved clinical responses, suggesting that efforts to enhance persistence of engineered T cells will result in improved clinical responses. This clinical success has forged many academic/non-profit partnerships with large pharmaceutical companies to address the challenge of converting the technology and infrastructure required to treat a small number of patients on a phase I clinical trial to a therapy that can be used worldwide to potentially treat up to many thousands of patients yearly.^6^ One of these challenges is that human serum is used to expand the genetically engineered T cells.^7^ Human serum is expensive; requires adventitious agent testing and could potentially contain emerging infectious agents; varies considerably from lot to lot, requiring frequent screening; and may contain agents harmful for T cell expansion and survival. Additionally, the current supply of human serum will not meet demand if more than one blockbuster T cell therapy is approved.^7^ Thus, a T cell manufacturing process that is not dependent on human serum would be an important step to make adoptive T cell therapy less expensive, more consistent, and available to more patients.

The first serum-free medium (SFM) was developed in 1965,^8^ and since then, several improved media have been introduced into the market. Arguably, the most commonly used medium for T cell expansion is RPMI 1640 supplemented with 10% fetal bovine serum.^9^ Extensive research to eliminate serum from cell culture media in the late 1970s led to the development of Iscove's modified Dulbecco's medium (IMDM), which added key components, such as human transferrin, complex lipids, and supplemental buffering capacity with HEPES to DMEM.^10^ A 1:1 volumetric mixture of DMEM and F-12 medium resulted in DMEM:F12, which, when supplemented with insulin, transferrin, selenium, and putrescine, was able to support robust cell expansion and clonal selection in the absence of serum.^11^ In the late 1980s and early 1990s, development of proprietary cell culture media for T cell expansion was based on modifications of both IMDM and DMEM:F12. Extensive modifications to DMEM:F12 gave rise to GIBCO AIM-V,^12^ whereas modifications to IMDM gave rise to the X-VIVO series of hematopoietic media.^13^ CTS OpTmizer SFM was later developed as a more robust medium for high-density T cell expansion in a perfusion bioreactor.^14^ There is no consensus on what is the best media to use for adoptive T cell therapy; however, most groups to date have used RPMI 1640,^15^, ^16^, ^17^ AIM V,^18^, ^19^, ^20^ or X-VIVO 15.^3^, ^21^, ^22^, ^23^, ^24^, ^25^ Both AIM V and X-VIVO 15 are defined as SFM, but in the T cell manufacturing process used to treat patients, human serum is universally added, largely because patient-derived T cells fail to grow optimally in serum-free media and exhibit reduced efficacies of gene transfer resulting from less than optimal T cell activation.^26^ Scarce new progress has occurred in defining improved media for *ex vivo* expansion of human T cells for adoptive T cell therapy because most experimental and commercial cell culture media for T cell expansion offer variations and modifications of these classical media.

Within the last several years, the field of immunometabolism has re-emerged to the forefront of immunology and much has been learned about how T cell metabolism affects T cell function.^27^, ^28^, ^29^ Glucose, glutamine, and serine are essential nutrients for T cell expansion and function.^30^, ^31^, ^32^ Metal ions (e.g., Ca^2+^ and Zn^2+^) are important cofactors for proteins and serve as intracellular signaling messengers.^33^ The media currently being used for adoptive T cell therapy does not benefit from the recent advances in understanding T cell metabolism. Given the importance of advanced cell culture systems for successful manufacturing of T cell therapies, we recently developed a fully defined medium that could expand all human T cell subsets in the absence of human serum. Here, we tested the ability of this serum-free media to expand T cells for adoptive T cell therapy. Not only did this these media robustly expand T cells from healthy donors and patients, we show that the addition of human serum hinders rather than supports T cell function *in vivo*. Our studies suggest that adoptive T cell therapies will be more effective if the T cells are cultured in serum-free media prior to infusion into patients.

## Results

### 1B2H Supports Robustly Expands Human T Cells in the Absence of Serum

**Figure 1:**
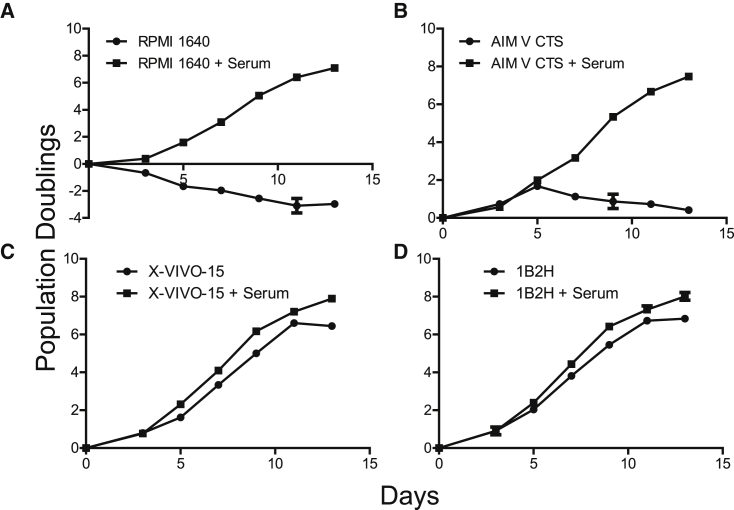
1B2H Supports and Robustly Expands Human T Cells in the Absence of Serum Primary human CD4 and CD8 T cells were mixed at a 1:1 ratio, activated with CD3/28-coated beads, and cultured in (A) RPMI, (B) AIM V CTS, (C) X-VIVO 15, or (D) 1B2H media with no serum (circles) and with serum (squares). The population doubling rate was measured by cell counting on the days indicated by a symbol. Error bars represent SEM.

We recently generated a fully chemically defined media (currently called 1B2H) that could expand naive (T_N_), central memory (T_CM_), and effector memory (T_EM_) equally well in the presence and absence of human serum (C.E., L. Guo, S. Voicu, L. Gil-de-Gómez, A.R.M., L. Cortina, J. Pajda, M. Andolina, M. Torres-Castillo, J. Donato, S. Mansour, E. Zynda, A.V.-R., I. Blair, and J.L.R., unpublished data). We wished to compare this medium, termed 1B2H, to media that is currently being used in adoptive T cell therapy trials. CD4 and CD8 T cells were isolated from healthy donors, activated with anti-CD3/28-coated beads, and cultured in RPMI 1640, AIM V, X-VIVO 15, and 1B2H, with and without serum. As expected, RPMI 1640 required the addition of human serum to promote T cell expansion (Figure 1A). T cells expanded in AIM V grew as well as T cells expanded with AIM V supplemented with human serum for 5 days. However, after 5 days, AIM V SFM could not support further T cell expansion (Figure 1B). In contrast, both X-VIVO 15 and 1B2H supported T cell expansion nearly equivalently in the presence and absence of serum, although it is important to note that T cells expanded in the absence of serum rested more quickly than T cells expanded in the presence of serum (compare T cell growth between day 12 and day 15; Figures 1C and 1D). Thus, both X-VIVO 15 and 1B2H fulfill the requirement of being a bona fide serum-free medium: similar expansion in the presence and absence of human serum.

### 1B2H Supports the Expansion of a More Differentiated T Cell Phenotype

**Figure 2:**
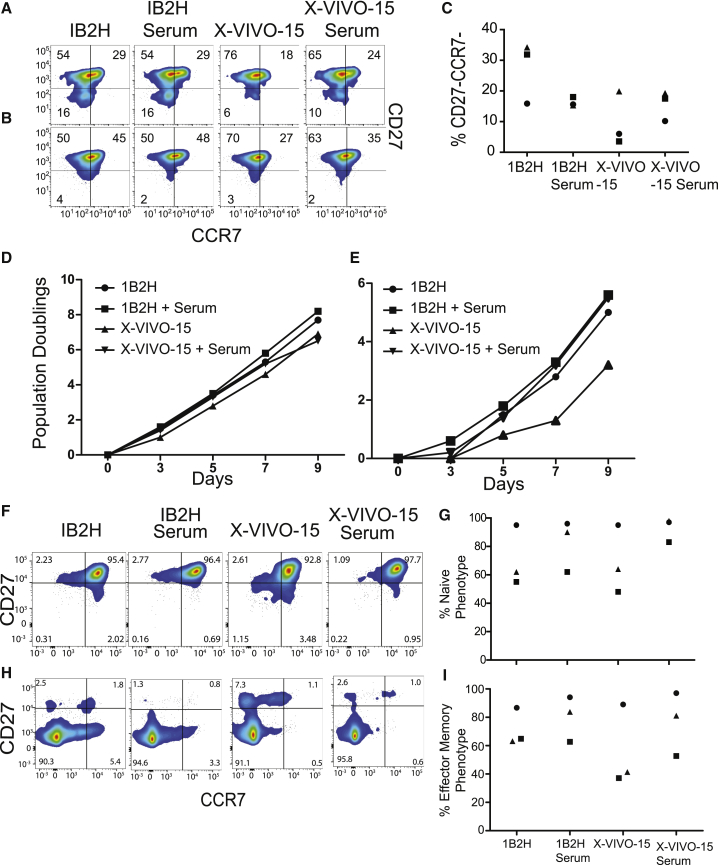
1B2H Supports the Expansion of a More Differentiated T Cell Phenotype (A and B) Expanded CD4 (A) and CD8 (B) T cells from Figure 1 were stained for CD27 and CCR7 by flow cytometry. (C) Summary data from three independent experiments showing the percentage of CD27^−^, CCR7^−^ T cells after T cell expansion. Each symbol represents a different donor. (D and E) Naive (CD45RA^+^, CCR7^+^, and CD27^+^) (D) and effector memory (CD45RO^+^, CCR7^−^, and CD27^−^) (E) were sorted and activated with CD3/28-coated beads in the indicated media, and cell expansion was monitored on the indicated days. (F–I) CCR7 and CD27 phenotype of naive, CCR7^+^, CCR7^+^, (F and G) and effector memory (H and I). T cells analyzed after 9 days of culture. (G) and (I) represent summary phenotype data from three independent experiments.

We examined the phenotype of T cells expanded in either IB2H or X-VIVO 15. Interestingly, 1B2H supported the expansion of CD4, and to a lesser extent CD8, T cells that did not express either CCR7 and/or CD27, hallmarks of T cells with a more differentiated or T_EM_ phenotype (Figures 2A–2C).^34^, ^35^, ^36^ One interpretation of this data is that 1B2H is more effective at expanding highly differentiated T cells and another interpretation is that less differentiated T cells expanded in 1B2H more rapidly convert into effector T cells. To distinguish between these possibilities, we sorted naive and T_EM_ CD4 T cells and expanded them in either 1B2H or X-VIVO 15 in the presence or absence of human serum. We observed that naive T cells expanded better in 1B2H media and the addition of human serum did not alter the growth of T cells in either media (Figure 2D). Effector T cells, on the other hand, did not proliferate well in X-VIVO 15 in the absence of serum but did expand in the presence of serum. In contrast, equivalent expansion of effector T cells in 1B2H occurred in the presence or absence of serum (Figure 2E). We also examined the phenotype of the expanded, purified T cell populations. Regardless of the medium, purified naive T cells maintained high CCR7 and CD27 expression, although T cells expanded in the presence of serum maintained these markers better than T cells expanded in the absence of serum (Figures 2F and 2G). T_EM_ T cells expanded in 1B2H with and without serum or in X-VIVO 15 with serum maintained their T_EM_ phenotype (Figures 2H and 2I), whereas a population of T_CM_ T cells were enriched in populations grown in X-VIVO 15 without serum, suggesting that some of the expansion observed in X-VIVO 15 without serum was due to contaminating T cells. Together, these data suggest that 1B2H can expand both naive and T_EM_ T cells in the absence of serum, whereas X-VIVO 15 can only expand naive T cells as a serum-free media. Moreover, T cells expanded with 1B2H maintain their initial T_EM_ phenotype better than T cells expanded with X-VIVO 15.

### T Cells Expanded in the Absence of Serum Are Amenable to Genetic Modification and Have Improved *In Vitro* Functional Activity

**Figure 3:**
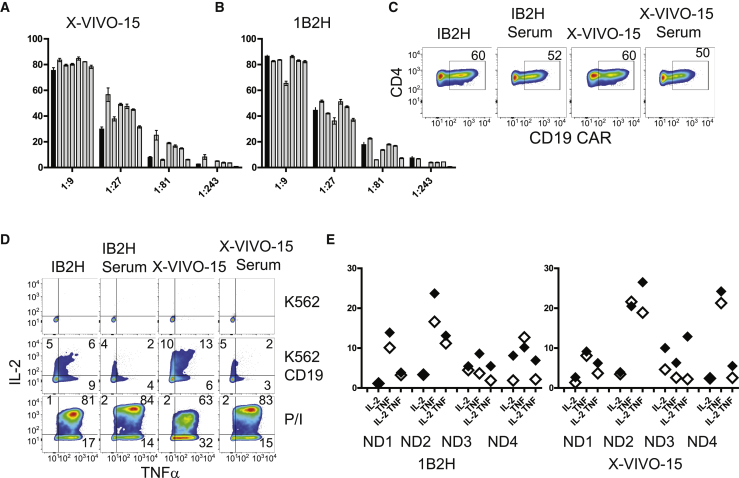
T Cells Expanded in the Absence of Serum Have Improved In Vitro Functional Activity (A and B) Human T cells were activated with CD3/28 beads in either X-VIVO 15 (A) or 1B2H (B). Media in the absence of human serum (black bar) or in the presence of 1 of 6 lots of human serum (white bars, with each bar representing a different lot of serum) and transduced with a GFP-expressing lentiviral vector at the indicated dilutions. 5 days after transduction, percentage of GFP positivity was determined by flow cytometry. Error bars represent SEM. (C) Primary human CD4 T cells were transduced with the CD19 CAR construct and percentage transduction was determined by flow cytometry after 10 days of culture. (D) The day after CD19 CAR staining, T cells from (C) were incubated with K562, CD19-expressing K562, or PMA and ionomycin for 5 hr, and production of intracellular IL-2 and TNF-α was measured by flow cytometry. (E) Summary data from the experiment described in (C) for 4 donors. Filled symbols represent T cells expanded in 1B2H, and open symbols represent T cells expanded in X-VIVO 15.

The ability to reprogram T cells through genetic modification is key to the clinical success of many adoptive T cell therapy protocols. To assess whether T cells expanded in 1B2H were amenable to lentiviral transduction, we stimulated T cells cultured in 1B2H or X-VIVO 15 in the presence or absence of 1 of 6 human serum lots, and then transduced the activated T cells with a range of dilutions of a GFP-expressing lentiviral vector. As expected, there was considerable variability in the transduction efficiency of T cells cultured in the presence of different lots of serum. However, in most cases, we observed a higher transduction efficiency in T cells cultured using X-VIVO 15 in the presence of human serum versus without serum (compare the 1:27 and 1:81 dilutions that are in the linear range; Figure 3A). In contrast, T cells cultured in 1B2H in the absence of serum were just as susceptible to lentiviral transduction as T cells grown in the presence of human serum (Figure 3B). To determine whether T cells grown in 1B2H have distinct functional properties from those grown in X-VIVO 15, with and without 5% human serum, we transduced T cells with a CAR construct that redirects T cells to CD19-positive cells,^37^ which has been used in several successful adoptive T cell therapy trials (Figure 3C).^3^, ^5^, ^22^ These CD19 CAR-transduced T cells were then incubated with K562 cells, K562 cells expressing CD19, or PMA + ionomycin, and then analyzed for intracellular interleukin-2 (IL-2) and tumor necrosis factor alpha (TNF-α) production. Although there was no reproducible difference between T cells expanded with X-VIVO 15 or 1B2H, it was clear that T cells expanded in the absence of serum had improved *in vitro* function than those grown with serum, both in terms of the number of T cells that produced a single cytokine and those that produced both cytokines (Figures 3D and 3E).

### Durable Tumor Control by CAR T Cells Expanded in the Absence of Human Serum

**Figure 4:**
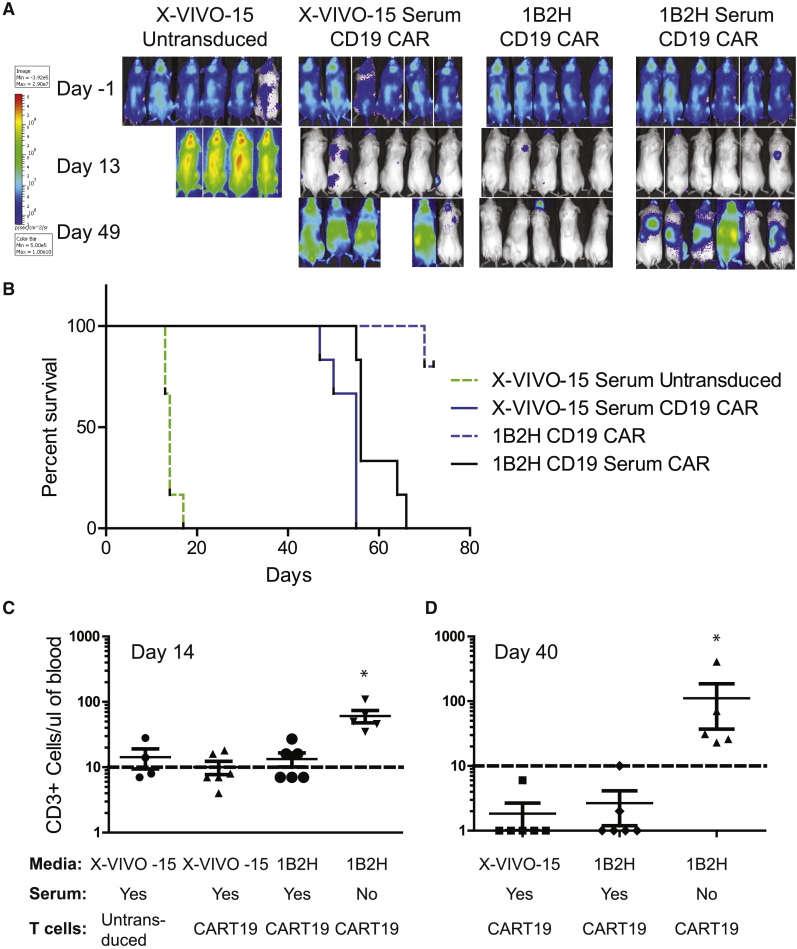
Durable Tumor Control by CAR T Cells Expanded in the Absence of Human Serum (A) NALM6 tumor cells expressing luciferase were engrafted in NSG mice, and 7 days later (day −1), luciferase expression was determined. Mice were then randomized into treatment groups and 10 million of the indicated T cells (described in Figure 3) were infused on day 0. Luciferase expression within each mouse was measured on days 13 and 43 after T cell infusion. (B) Kaplan-Meier curve showing tumor-free survival of each of the indicated groups. (C and D) 14 (C) and 40 (D) days after T cell infusion, peripheral T cell levels were measured using TruCount beads. Each symbol represents data collected from a single mouse.

An *in vivo* model of CAR T cell therapy was developed, which has proven informative for predicting clinical results in humans.^3^, ^21^, ^22^, ^37^, ^38^, ^39^, ^40^, ^41^ In this model, NALM6, a pre-B cell leukemic cell line that naturally expresses CD19, was engineered to express luciferase and engrafted into NOD/SCID-γc^−/−^ (NSG) mice. If CD19-specific CAR T cells are added within a week, tumor clearance is observed within 10–14 days. However, if the experiment is extended, tumor returns in most animals and durable cures are not observed. We sought to determine whether T cells expanded in 1B2H could perform *in vivo* as well as T cells expanded using X-VIVO 15 supplemented with 5% human serum—the current medium formulation our group uses for good manufacturing practice (GMP) manufacturing of T cells. Consistent with previous findings,^37^, ^38^, ^39^, ^40^ we observed that CD19-specific CAR T cells expanded in X-VIVO 15 supplemented with 5% human serum dramatically cleared tumors from NSG mice 13 days after the infusion of T cells (Figure 4A). However, as previously demonstrated, this control was transient, with tumor returning to most of the mice. Surprisingly, CD19-specific CAR T cells expanded in 1B2H SFM could provide durable control of tumors and this translated to a remarkable difference in leukemia-free survival (Figure 4B). Interestingly, mice treated with CAR T cells expanded with 1B2H supplemented with 5% human serum could only transiently control tumor burden similarly to mice treated with CAR T cells expanded in X-VIVO 15 supplemented with 5% human serum, suggesting that expanding T cells in the presence of serum is detrimental to *in vivo* performance of engineered T cells. Both humanized mouse studies and clinical data from patients have shown a strong correlation with the level of T cell persistence after adoptive transfer and the ability to provide durable control of tumors.^3^, ^37^ Likewise, we observed superior T cell engraftment that was sustained for much longer in cells expanded in the absence of serum (Figure 4C). Together, these data suggest that human serum is not only dispensable, but is also hindering the ability of CAR T cells to function, persist, and control tumor growth.

### 1B2H SFM Can Robustly Expand T Cells from Multiple Myeloma Patients

**Figure 5:**
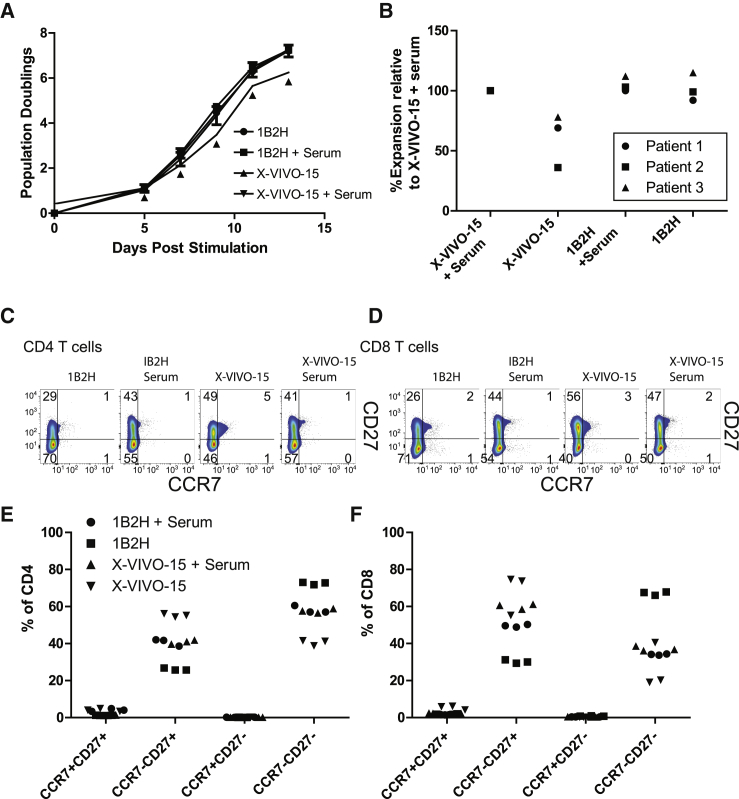
1B2H SFM Can Robustly Expand T Cells from Multiple Myeloma Patients (A) T cells from de-identified multiple myeloma patients were purified from frozen apheresis material, stimulated by CD3/28-coated beads, and expanded in the indicated media. Error bars represent SEM for one donor performed in triplicate. (B) Summary expansion data from 3 independent patients. Fold expansion of T cells cultured in X-VIVO 15 was set as 100%. (C and D) At the end of expansion, CD4 (C) and CD8 (D) were stained for CCR7 and CD27. (E and F) Summary data for 3 independent experiments showing the percentage of CCR7^+^CD27^+^, CCR7^+^CD27^−^, CCR7^−^CD27^+^, and CCR7^−^CD27^−^ in CD4 (E) and CD8 (F). Each symbol represents a different donor.

T cells from cancer patients are generally more differentiated and more challenging to expand than T cells from healthy donors. Because we observed that 1B2H expanded differentiated T cells better than X-VIVO 15, we wished to determine whether 1B2H could more efficiently expand highly differentiated T cells isolated from multiple myeloma patients. We repeated our T cell expansion studies using cryopreserved, de-identified apheresis products from multiple myeloma patients involved in a previous adoptive T cell therapy clinical trial.^42^ In contrast to data shown in Figure 1A, in which healthy donor T cells were studied, we observed that X-VIVO 15 supplemented with human serum expanded patient T cells better than T cells expanded in X-VIVO 15 SFM. Interestingly, we did not see this difference in T cells expanded using 1B2H (Figures 5A and 5B), indicating that 1B2H is a SFM for both healthy and multiple myeloma patient T cells. Like the healthy donors (Figure 2), 1B2H supported the expansion of highly differentiated T cells, with ∼70% of the T cells having the CCR7^−^, CD27^−^ phenotype (Figures 5C–5F). These data suggest that the ability of 1B2H to expand patient T cells equally well in the presence and absence of serum is linked to its ability to expand T cells with a more differentiated phenotype.

### Durable Control of Tumor Growth of Patient T Cells Expanded with 1B2H SFM

**Figure 6:**
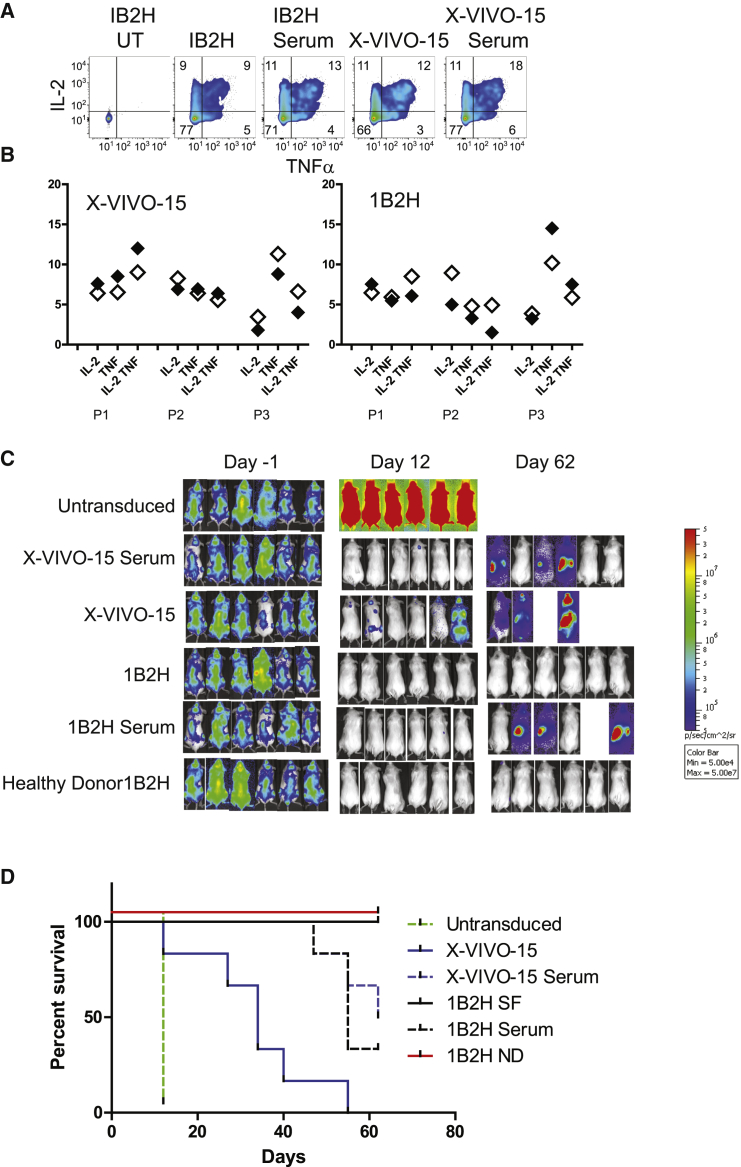
Durable Control of Tumor Growth of Patient T Cells Expanded with 1B2H SFM (A) CD19 CAR transduced T cells from Figure 5 were diluted with untransduced (UT) T cells so that each population contained 40% transduced T cells. These cells were then incubated with CD19-expressing K562 for 5 hr, and intracellular IL-2 and TNF-α were measured by flow cytometry. (B) Summary data for 3 multiple myeloma patients from the data described in (A). Filled symbols represent T cells expanded in serum, and open symbols represent T cells expanded in the indicated media in the absence of serum. (C) NALM6 tumor cells expressing luciferase were engrafted in NSG mice, and 7 days later (day −1), luciferase expression was determined. Mice were then randomized into treatment groups and 10 million of the indicated T cells (described in Figure 5) were infused on day 0. Luciferase expression within each mouse was measured on days 12 and 62 after T cell infusion. (D) Kaplan-Meier curve showing tumor-free survival (animals that maintained less than the 1 × 10^7^ light units) of each of the indicated groups.

We also examined the functional properties of the multiple myeloma patient T cells expanded in X-VIVO 15 or 1B2H, with and without serum. Here, the addition of serum did not significantly alter the functional profile of the expanded CD19-specific CAR T cells because there were no consistent differences between T cells grown in 1B2H or X-VIVO 15 (Figures 6A and 6B), suggesting the functional abilities of fully differentiated T cells are not modulated by the presence of human serum or media in which they are expanded. We then addressed the ability of these expanded T cells to control NALM6 tumors *in vivo*. As a control, we also expanded CD19-specific T cells from a healthy individual so we could compare *in vivo* function of T cells from healthy donors and multiple myeloma patients. All T cell populations showed transient control of NALM6 tumors; however, 2 of the 6 mice that were treated with T cells expanded by X-VIVO 15 SFM still had significant tumor burden after 12 days of treatment (Figure 6C) and none of these mice durably controlled tumor growth (Figure 6D). As before, the leukemia relapsed in most mice treated with CD19-specific T cells expanded in either 1B2H or X-VIVO 15 supplemented with human serum. Impressively, T cells isolated from multiple myeloma patients engineered to be CD19 specific and expanded in 1B2H SFM provided durable control of tumor in all mice treated and had equivalent therapeutic potency as similarly engineered and expanded T cells isolated from a healthy donor.

## Discussion

Adoptive T cell therapy using genetically engineered T cells is an emerging treatment option that has shown great promise to become the standard of care for many B cell-derived tumors.^43^ Additionally, much effort is currently underway to increase the number of tumors that can be targeted by this approach as well as to expand beyond cancer into other indications, such as new treatments for infectious and autoimmune diseases.^44^, ^45^, ^46^ The transition from a boutique therapy to one that could potentially treat tens of thousands of patients a year is not trivial. However, the fruits of this scale-up process will be a T cell engineering process that is more efficient, less labor intensive, and less expensive. Additionally, the increased demand for equipment and reagents required to manufacture T cells for therapy will create competition and innovation in the field, further reducing costs and increasing both efficiency and consistency of the manufacturing process. However, the current practice of using human serum to supplement media for *ex vivo* T cell expansion will be difficult, if not impossible, to scale-up to meet demand. All adoptive T cell therapy trials to date have employed human serum due to the difficulty of expanding T cells from patients. Supplementing media with human serum has been feasible to do for proof of principle studies up to phase III trials; however, as more therapies near US Food and Drug Administration (FDA) approval, it is highly likely that human serum will become scarce and, unless radical measures are employed to increase the supply of human serum, it will be become a rate limiting reagent for adoptive T cell therapy trials.

Both our new medium 1B2H and X-VIVO 15 could expand T cells from healthy donors equally well in the presence and absence of serum. Surprisingly, we found that the addition of human serum decreased T cell functionality *in vitro* and *in vivo*. T cells engineered to express a CD19-specific CAR expanded in the absence of human serum durably controlled leukemia, whereas the same T cells expanded in the presence of serum could only confer transient control of tumor growth. This is a striking finding that provides the basis and rationale to perform a clinical trial in humans to determine whether T cells expanded in the absence of serum have a greater therapeutic effect than those expanded with human serum. Human serum is complex.^47^, ^48^ It is difficult to ascertain which components fortify media such as RPMI 1640 and AIM-V to allow them to expand T cells and which components of serum hinder T cell function and the ability to engraft and persist. Nonetheless, our data demonstrate that serum is a double-edged sword: for non-optimized media, it provides factors required for expansion, but at the price of reduced functionality and *in vivo* function. Our data clearly show that expanding T cells in the absence of human serum improves both the functionality and durability of engineered T cells and cautions against the continued use of human serum in the clinical manufacturing of T cells for use in adoptive T cell therapy.

With T cells isolated from healthy donors, we observed that 1B2H and X-VIVO 15 could equivalently expand T cells. However, our data show that 1B2H medium better enabled T cell transduction and expanded T cells with a more differentiated phenotype. This ability of 1B2H to expand highly differentiated T cells was most apparent when T cells isolated from multiple myeloma patients were studied. Here, 1B2H could expand these cells, whereas X-VIVO 15 could not. At this point, it is difficult to pinpoint exactly why 1B2H expands effector T cells better than X-VIVO 15. The precise formulations of both of these media are proprietary, so a side-by-side comparison is not possible. Moreover, it is likely that multiple factors are responsible for this difference, so it would be quite arduous to precisely define what enables the effector T cells to grow better in 1B2H, even if the exact formulations were known. Because 1B2H expanded all T cell populations after sorting better than X-VIVO 15 (Figure 2D), it is possible that 1B2H is better suited to support expansion of differentiated and/or stressed T cells.

Although there is no consensus on what is the best T cell to use for adoptive T cell therapy, there is a growing appreciation that less differentiated T cells are better suited to provide durable control of tumors than more differentiated T cells.^49^, ^50^, ^51^ Our findings do not dispute this notion. Rather, our results indicate that if a patient has largely T_EM_ T cells, the use of optimized media such as 1B2H will enhance the expansion of these T cells in the absence of human serum, which could reduce the number of individuals that are unable to receive this therapy due to poor *ex vivo* T cell expansion. Moreover, by expanding these T cells in SFM, our data would predict that more of these individuals would have durable anti-tumor responses. Thus, use of 1B2H media to expand T cells for adoptive T cell therapy should enable more patients to receive this therapy and more of these individuals should experience durable remissions.

## Materials and Methods

### T Cell Isolation

De-identified, purified human CD4 and CD8 T cells were obtained from the Human Immunology Core of the University of Pennsylvania under an institutional review board (IRB)-approved protocol. De-identified frozen apheresis bags from multiple myeloma patients were provided by Dr. Nicole Aqui. After thawing, CD4 and CD8 T cells were purified using RosetteSep (Cat #15062 and 15063) per the manufacturer’s recommendations.

### Media Preparation

RPMI 1640 (Thermo Fisher Scientific, Cat #11875-085), AIM V (Thermo Fisher Scientific, Cat #0870112DK), or X-VIVO 15 (Lonza, Cat #04-744Q) and 1B2H containing 17.5 mM glucose and 17.5 galactose (described by Ecker et al.) were supplemented with N-acetyl cysteine, 4.6 g/L, and Normocin (InvivoGen) 100 μg/mL. Where indicated, human serum (Thermo Fisher Scientific) was used at 5% final volume.

### Lentiviral Production

Production of lentiviral vectors expressing CD19-4-1BB-zeta^37^ and GFP^52^ has previously been described.

### T Cell Activation, Transduction, and Expansion

Unless indicated otherwise, CD4 and CD8 T cells were mixed at a 1:1 ratio, placed in the indicated media, and stimulated using anti-CD3- and anti-CD28-coated beads (Thermo Fisher Scientific, Cat# 1141D). After 24 hr, T cells were transduced with concentrated lentiviral supernatant as previously described.^53^ T cells were counted every other day starting on day 3, and cell size was monitored on a Multisizer 3 Coulter Counter (Beckman Coulter, Indianapolis, IN). After counting, cells were diluted to 500,000 per mL using fresh media. Cells were cultured until their mean cell volume approached 250 fl and they stopped accumulating.

### Flow Cytometry and Intracellular Cytokine Staining

CD27 (BioLegend Cat #302830) CCR7 (BioLegend Cat #353218) labeled antibodies were used to stain the expanded T cell populations as previously described.^54^ Expression of the CD19 CARs was detected using the biotinylated F(ab′)2 fragment from goat anti-mouse immunoglobulin G (IgG) sera (specific for scFvs of murine origin; Jackson ImmunoResearch), followed by staining with streptavidin-PE (BD Biosciences/Pharmingen, Cat# 554061). Intracellular cytokine staining was performed as previously described.^55^ Briefly, after T cells stopped expanding, CD3/28 beads were removed by magnetic separation. The following day, the T cells were mixed with K562s expressing CD19 at a ratio of 2:1 and incubated for 5 hr with GolgiStop (BD Biosciences). Surface CD4 (BioLegend Cat# 317433) and CD8 (BD Biosciences Cat# 560273) staining was performed, samples were fixed, and the following day, intracellular staining was performed as previously described,^56^ with an LSR II flow cytometer (BD Biosciences, San Jose, CA) and FlowJo software (Tree Star, Ashland, OR). The following antibodies were used for intracellular staining: -IL-2 (BD Biosciences Cat# 554567), interferon γ (IFNγ) (BD Biosciences Cat# 552882, TNF (BD Biosciences Cat# 557647), and MIP-1β (BD Biosciences Cat# 560688).

### *In Vivo* Humanized Mouse Studies

6- to 10-week-old NSG mice were obtained from Jackson Laboratory (Bar Harbor, ME) or bred in-house under an approved institutional animal care and use committee protocol and maintained under pathogen-free conditions. Animals were injected via tail vein with 1 million viable NALM6 cells (ATCC CRL-3273) engineered to express click beetle green (CBG) luciferase. 8 days after injection of leukemic cells, mice received 10 million T cells via tail vein injections. Mice were monitored weekly using bioluminescent imaging to determine the presence of tumor using the Xenogen Spectrum (Caliper Life Sciences, Hopkinton, MA) imaging system using Living Image v4.2 software, as previously described.^39^ Peripheral blood was obtained by retro-orbital bleeding, and blood was examined for evidence of leukemia and T cell engraftment by flow cytometry using BD Trucount (BD Biosciences, Cat #340334) tubes as previously described.^52^

### Statistical Analysis

Survival analysis, SEM, t test, and ANOVA were performed using GraphPad Prism software (GraphPad, La Jolla, CA)

## Author Contributions

A.R.M., C.E., H.K., E.A.W., and J.G. were involved in conducting the research; A.R.M., C.E., H.K., E.A.W., A.V.-R., and J.L.R. were involved in preparation of the data for publication; A.R.M., C.E., E.A.W., H.K., A.V.-R., and J.L.R. reviewed and edited the manuscript; A.V.-R. and J.L.R. were involved in the conceptualization, funding acquisition, project administration, and writing of the original draft.
